# Comparing a Mobile Decision Support System Versus the Use of Printed Materials for the Implementation of an Evidence-Based Recommendation: Protocol for a Qualitative Evaluation

**DOI:** 10.2196/resprot.9827

**Published:** 2018-04-13

**Authors:** Jhon Camacho, Ana María Medina Ch., Zach Landis-Lewis, Gerald Douglas, Richard Boyce

**Affiliations:** ^1^ Departamento de Epidemiología Clínica y Bioestadística Pontificia Universidad Javeriana Bogotá Colombia; ^2^ Department of Biomedical Informatics University of Pittsburgh Pittsburgh, PA United States; ^3^ Instituto de Envejecimiento Pontificia Universidad Javeriana Bogotá Colombia; ^4^ Department of Learning Health Sciences University of Michigan Ann Arbor, MI United States

**Keywords:** practice guideline, implementation science, decision support systems, mhealth, technology acceptance, computer-interpretable clinical guidelines, Colombia

## Abstract

**Background:**

The distribution of printed materials is the most frequently used strategy to disseminate and implement clinical practice guidelines, although several studies have shown that the effectiveness of this approach is modest at best. Nevertheless, there is insufficient evidence to support the use of other strategies. Recent research has shown that the use of computerized decision support presents a promising approach to address some aspects of this problem.

**Objective:**

The aim of this study is to provide qualitative evidence on the potential effect of mobile decision support systems to facilitate the implementation of evidence-based recommendations included in clinical practice guidelines.

**Methods:**

We will conduct a qualitative study with two arms to compare the experience of primary care physicians while they try to implement an evidence-based recommendation in their clinical practice. In the first arm, we will provide participants with a printout of the guideline article containing the recommendation, while in the second arm, we will provide participants with a mobile app developed after formalizing the recommendation text into a clinical algorithm. Data will be collected using semistructured and open interviews to explore aspects of behavioral change and technology acceptance involved in the implementation process. The analysis will be comprised of two phases. During the first phase, we will conduct a template analysis to identify barriers and facilitators in each scenario. Then, during the second phase, we will contrast the findings from each arm to propose hypotheses about the potential impact of the system.

**Results:**

We have formalized the narrative in the recommendation into a clinical algorithm and have developed a mobile app. Data collection is expected to occur during 2018, with the first phase of analysis running in parallel. The second phase is scheduled to conclude in July 2019.

**Conclusions:**

Our study will further the understanding of the role of mobile decision support systems in the implementation of clinical practice guidelines. Furthermore, we will provide qualitative evidence to aid decisions made by low- and middle-income countries’ ministries of health about investments in these technologies.

## Introduction

### Printed Materials for the Dissemination and Implementation of Clinical Practice Guidelines

Successful implementation of clinical practice guidelines (CPGs) has the potential to reduce health care costs and improve the quality of care by promoting the use of cost-effective, evidence-based interventions [1-3]. However, the extent of these benefits varies largely across implementation sites [1], and even between recommendations in the same guideline [2]. This variability could be attributed, in part, to the way CPGs are disseminated and implemented [1,4].

Distribution of printed materials has been the predominant CPG dissemination and implementation (D&I) strategy [2,3]. In 2004, Grimshaw et al [1] conducted an extensive literature review about the effectiveness of guideline D&I strategies. Among the studies evaluating the sole distribution of printed materials, the authors found that although most reports presented improvements in dichotomous process variables (eg, the proportion of patient encounters that follow the recommendation), the median effects were modest (8.1%, range 3.6% to 17.0%). Eight years later in 2012, literature reviews from Brusamento et al [5] and Giguère et al [6] found that this situation had not changed significantly.

### Decision Support Systems for the Implementation of Clinical Algorithms

Some evidence-based recommendations ask the clinicians to implement clinical algorithms. In these cases, decision support systems (DSSs) provide an approach that facilitates the understanding of the recommendation among the intended users and its integration into their daily routine [7]. In 2005, Garg et al [8] conducted a review of trials evaluating the effect of DSSs in changing clinical practice. The authors found that these systems improved practitioner performance in 64% of the cases reported. Based on these results, Garg et al [8] highlighted the need for further research into the determinants of DSS acceptance and overall success.

Recent research has shown that it is feasible to use DSSs to support the implementation of clinical algorithms, even in resource-constrained environments [9,10]. Nevertheless, before these systems can be widely used as a D&I strategy, it is necessary to solve two informatics challenges.

The first of these challenges is that CPG recommendations are often unclear, ambiguous or incomplete [7,9,11-13], making it difficult to transform them into decision algorithms. This problem can be addressed through formalization processes that translate the recommendation into specific tasks and decision procedures while allowing for the identification of areas where the recommendations are ambiguous or evidence is missing [7].

The second challenge is that in many cases, DSSs rely on their integration into other clinical information systems, or at least on the availability of personal computers. This dependency on informatics infrastructure represents a barrier to the broad application of these systems as a D&I strategy. Most care centers in low-income countries do not have personal computers, let alone clinical information systems. Even in the case of middle-income countries, rural areas frequently present the same general shortage of computer infrastructure. Furthermore, although it is typical that urban care centers in middle-income countries and other technology-rich environments have electronic health records, these are often homegrown or produced by many different vendors. Consequently, the broad integration of DSSs into these systems would imply enormous investments, requiring the modification of many programs and coordination among many parties.

### Mobile Decision Support Systems

Recent evidence suggests that mobile technologies could provide an approach to address these barriers, allowing DSSs to be implemented without requiring personal computers or its integration into other clinical information systems [9,14,15]. However, there is insufficient evidence supporting the effectiveness of these technologies [16,17]. This is in part due to a shortage of formal evaluations [18]. The authors of recent literature reviews have highlighted the need for theory-based research about the factors that may influence the adoption and scalability of these interventions [19], particularly those aimed at promoting practice changes [20].

### Objective

Our goal is to provide qualitative evidence on the potential effect of mobile DSSs to facilitate the implementation of evidence-based recommendations, as well as the determinants of their adoption. To achieve this aim, we will compare the experience of primary care physicians while they try to implement a recommendation in their clinical practice using either a printout of the journal article containing the recommendation or a mobile DSS. This comparison will consider aspects related to the behavioral change intended and those related to the acceptance of each technology.

## Methods

### Overall Design

**Figure 1:**
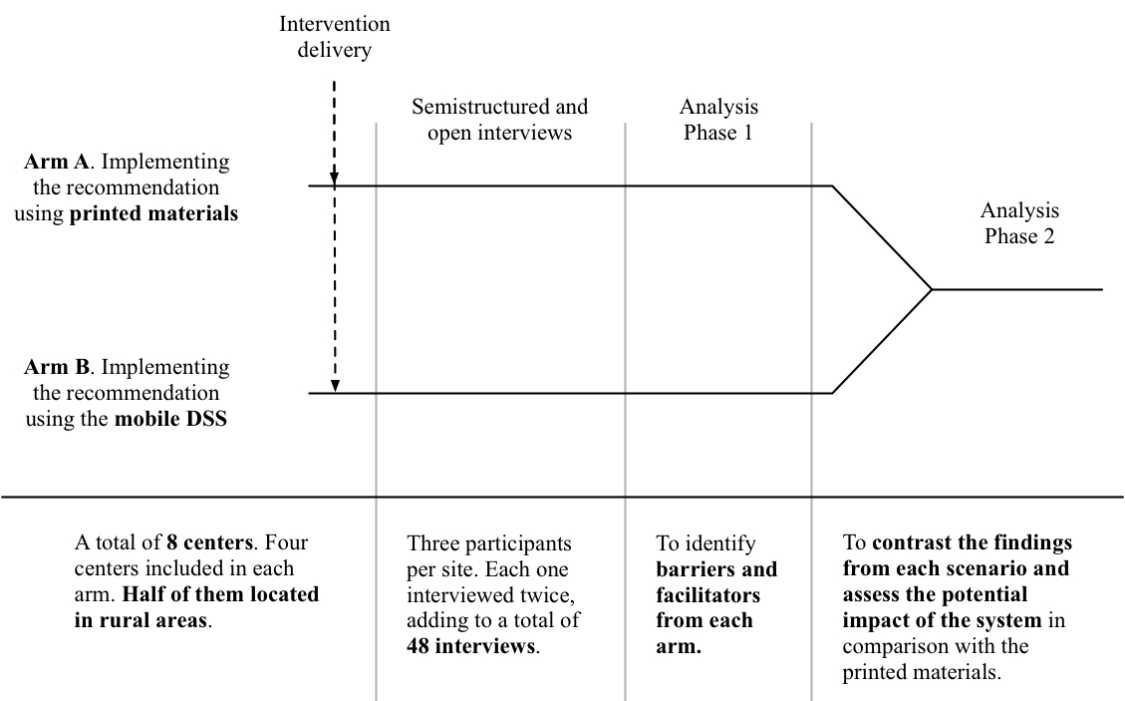
Study design. DSS: decision support system.

As shown in Figure 1, we will conduct a qualitative study with two arms. Participants will be asked to try to implement an evidence-based screening recommendation in their daily practice as primary care physicians, after being provided with either a journal article or a mobile DSS.

Arm assignment will be by center. Therefore, all participants from the same center will receive the same intervention. We will include a minimum of eight centers, half of them located in rural areas. We will assign centers to each arm iteratively as they enter the study, ensuring that half of the urban centers, as well as half of the rural centers, will be assigned to each arm.

Data will be collected through qualitative interviews, starting 1 month after the subjects received the article or the mobile app. During this time, the subjects will try to implement the screening recommendation during their daily practice as primary care physicians.

Analysis will focus on identifying barriers and facilitators for the implementation and contrasting these findings between the two arms.

### Study Case

We will ask participants to try to implement the case finding recommendation included in the Colombian clinical practice guideline for chronic obstructive pulmonary disease. This recommendation instructs the physician to check a series of risk factors, signs, and symptoms in adults, 40 years or older. When the suspicion is established, the guide recommends ordering a spirometry test to confirm or rule-out the diagnosis [21].

The selection of this recommendation was arbitrary. The only criteria considered was that it included a clinical algorithm (in this case, the decision about ordering a spirometry test based on a series of risk factors, signs, and symptoms) and that it would be part of a Colombian CPG.

### Interventions

**Figure 2:**
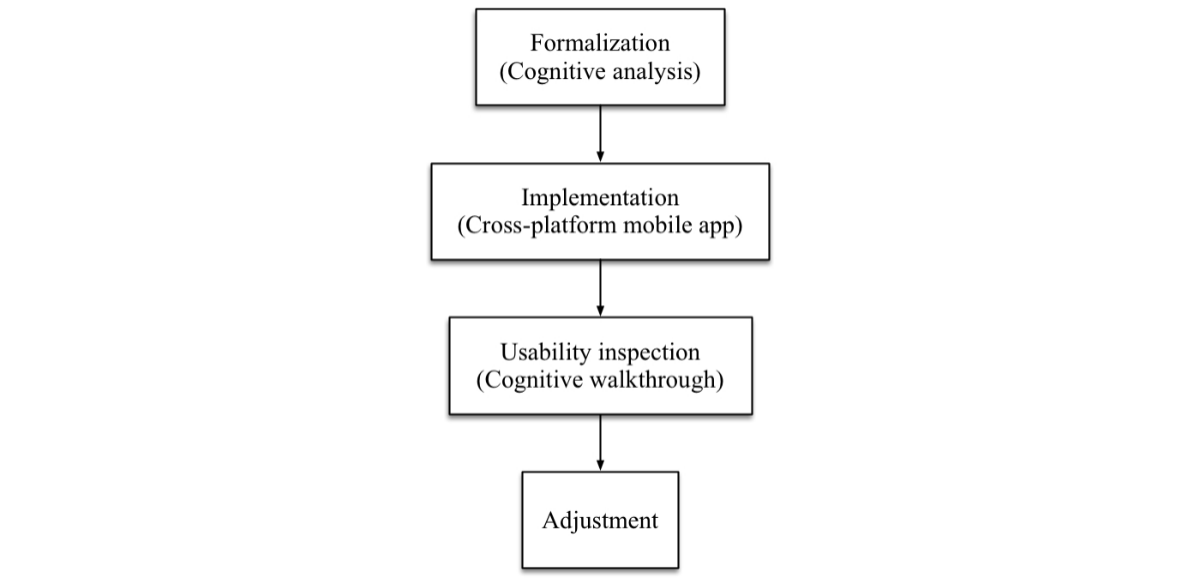
Stages of the system development.

Participants from centers assigned to arm A will be provided with a printout of the journal article in which the guideline was published [21]. Participants from centers assigned to arm B will be provided with a DSS implemented as a mobile app. As shown in Figure 2, the system will be developed following a process comprising four stages.

During the first stage, we will conduct a series of meetings with the guideline developers. Throughout these sessions, we will apply the cognitive analysis proposed by Patel et al [22] to formalize the recommendation into a clinical algorithm. Using this method, we will identify and encode the propositions stated in the text, and develop a conceptual model of the knowledge expressed. Throughout this process, we will identify and correct areas where the instructions allow different interpretations, the understanding depends on tacit knowledge, or the information is incomplete.

During the second stage, we will implement a mobile app that will support the participants in the implementation of the clinical algorithm. The app will be responsible for asking the user for information related to risk factors, signs, and symptoms, making calculations, and explaining the algorithm’s end points. To improve the chances of acceptance, the app will be able to run on Android, iOS, and Windows Mobile devices, and will operate autonomously, without the need for internet connectivity. The latter requirement will ensure that participating in the study will not generate new or unexpected costs for our subjects.

During the third stage, we will inspect the usability of the app prototype by conducting a cognitive walkthrough [23,24]. This method will identify aspects of the app that could hinder its use. Two recently graduated physicians and a mobile app developer will attend the session, and the principal investigator will act as the facilitator. Finally, during the fourth stage, the findings from the cognitive walkthrough will be used to improve the app.

### Selection of Sites and Participants

Our study will include a minimum of eight health centers, and a minimum of three participants per center, for a minimum sample of 24 participants. Half of the centers will be assigned to each arm.

We will conduct the study in Colombia. To explore the influence of the availability of informatics resources, half of the centers will be in Bogotá, while the other half will be in rural areas. The Bogotá sites will operate in a technology-rich environment that includes the use of clinical information systems and access to the internet during patient visits. In contrast, the rural sites will fulfill the criteria of having neither clinical information systems nor access to the internet during patient visits.

Our subjects will be general practitioners operating a regular primary care practice with adult patients.

### Intervention Delivery

At each site, the study will start with a short talk with the subjects. During this meeting, the principal investigator will outline the study procedures, and 1 of the guideline developers will present the screening recommendation, including a summary of the literature supporting it. At the end of the meeting, we will provide the participants assigned to arm A with a printout of the journal article in which the practice guideline was published [21]. In sites assigned to arm B, we will demonstrate how to install the app, and if necessary, help the subjects until they have the system running on their phones. No other explanation about the app will be provided. Not having explained how to use the app, we expect to be able to explore aspects of the system’s learnability [25]. In the case that a participant does not own an Android, iOS or Windows Mobile phone, we will provide them with a smartphone.

### Recruitment

To gain access to the sites, we will arrange meetings with their directors. During the meeting, we will present the project, ask for permission to recruit participants among the primary care physicians, ask for the director’s help in the recruitment process, and collect information about previous initiatives to implement CPGs, and the number of potential participants and their schedule.

The general practitioners from each center will be invited to participate by email (if possible, from the director of the respective site). The email will contain a description of the research goals, an outline of the study procedures (eg, the participant’s attempt to implement the screening recommendation), the informed consent document, and contact information. The email will also ask the potential participant to respond, either by email or phone, to express their decision to participate or not. Two weeks after sending the email, we will visit potential participants who have not responded, to invite them personally.

To incentivize participation, potential subjects will be offered with two COP $100,000 prepaid cards from a local supermarket (worth approximately US $35).

### Conceptual Framework

Our data collection and analysis will explore factors influencing the behavioral change intended by the recommendation, as well as those that regulate the system’s acceptance among the intended users. To achieve this, we will develop a conceptual framework that will harmonize the Theoretical Domains Framework (TDF) [26,27], the Unified Theory of Acceptance and Use of Technology (UTAUT) [28], and constructs from recent literature reviews about the acceptance of mobile health (mHealth) systems and DSSs.

To harmonize the contributing frameworks, we will compare the constructs’ descriptions. Based on these comparisons, we will define the harmonized constructs using the following strategies:

1. We will unify the name of constructs that refer to very similar concepts. Example: both the TDF and the UTAUT consider the extent to which the context provides support and resources to accomplish the behavior or to use the system effectively. However, the TDF calls this construct “environmental context and resources,” while the UTAUT calls it “facilitating conditions.”
2. We will include constructs that we consider relevant to understand the determinants of behavioral change and technology acceptance, but are not included in all contributing frameworks. Example: a recent literature review from Khong et al [29] identified “threats to professional autonomy” as a determinant of the acceptance of DDSs. It could be argued that this construct could be considered part of the UTAUT’s “performance expectancy.” However, it could be useful to include this construct in the final framework specifically.
3. We will map constructs that we consider too specific to more abstract constructs included in other frameworks. Example: the same review from Khong et al [29] includes “usability” and “computer experience or computer skill” as determinants for DSS acceptability. These two constructs could be mapped to the UTAUT’s “effort expectancy.” Therefore, it could be argued that it would not be necessary to include them in the final framework.

The decisions about using strategies 2 and 3 will consider the usefulness of the resulting constructs in developing questions for data collection and their relevance in the context of the project’s scope.

#### Contributing Frameworks

##### Determinants of Behavioral Change

The TDF was proposed by Michie et al [26] in 2005 to organize theoretical constructs included in classic psychological theories about behavior change and make them more accessible to implementation researchers from other fields. In 2012, Cane and Michie [27] produced a revised version of the TDF, containing 84 determinants of behavioral change, grouped in 14 domains: Knowledge, Skills, Social/Professional Role and Identity, Beliefs about Capabilities, Optimism, Beliefs about Consequences, Reinforcement, Intentions, Goals, Memory, Attention and Decision Processes, Environmental Context and Resources, Social Influences, Emotions, and Behavioral Regulation.

##### Determinants of Technology Acceptance

In 2003, Venkatesh et al [28] reviewed and combined the eight predominant models at the time, to integrate the fragmented theory on technology acceptance. The resulting theory (ie, UTAUT) states that the regular use of a technology is determined by the performance gain the user expects to obtain (Performance Expectancy), the level of effort they expect using the system will demand (Effort Expectancy), the influence of important others (Social Influence) and the level of support they will obtain (Facilitating Conditions). The first three influence the user’s intention to use the tool, whereas the last modulates the actual use, independently of the intention.

UTAUT’s constructs are general enough to be applied to any technology. However, to focus our research, we will also include specific concepts that have been identified as influencing the acceptance of mobile and decision support systems.

##### Determinants of the Acceptance of mHealth Systems

In 2016, Gagnon et al [30] published a systematic review of the literature reporting factors that modulate health care professionals’ acceptance of mHealth systems. The authors identified 49 barriers and facilitators. Some examples of these determinants are: interoperability, design and technical concerns, physician salary status and reimbursement, and support and promotion of mHealth by colleagues [30].

##### Determinants of the Acceptance of Decision Support Systems

Finally, in 2015, Khong et al [29] published a systematic literature review about factors affecting the adoption of clinical decision support systems. They recognized 42 determinants, including: patient clinical status, fitness of task, credibility of system, and patient-user’s relationship.

### Data Collection

Data will be collected through qualitative interviews, starting 1 month after the intervention delivery. To leverage the conceptual framework, we will conduct semistructured interviews, which have a loose structure based on a set of open-ended questions that define the area to be explored, but allows divergence to explore new ideas or gather more detail [31]. However, to address the potential bias derived from the use of a predefined set of constructs, we will also conduct in-depth interviews [31], which will start with an open question about the subject’s experience implementing the recommendation using either the app or the journal article. From this point on, participant’s answers will dictate the course of the interview.

Each participant will be interviewed twice, the first interview concerning aspects of behavioral change, while the second, focused on aspects of technology acceptance. Before each interview round, the participants from the respective site will be assigned at random to attend a semistructured or in-depth interview. All interviews will be recorded and transcribed verbatim.

### Analysis Plan

The analysis will be comprised of two phases. During phase one, we will perform a template analysis [32], based on the conceptual framework, to identify barriers and facilitators to the implementation in each scenario. This technique is especially useful to leverage an extant conceptual framework in the thematic analysis of qualitative data. In template analysis, the researcher starts with an a priori code book (or template) representing topics to look for in the data. This code book is refined during the analysis, to allow for the inclusion of new topics, the removal of codes that prove to be unnecessary, and the reorganization of codes to reflect the importance of specific concepts [32]. We will conduct this phase in parallel with the data collection, allowing for the adjustment of the interview guides according to preliminary results.

Coding will be conducted independently by 2 researchers. We will use NVivo version 11 (QSR International) to support analysis and data management. Consensus meetings will be held after coding every 4 transcripts. Before each meeting, the intercoder agreement for each transcript will be assessed using the Coding Comparison tool provided by NVivo. During the meeting, the researcher will discuss changes to the coding template and review transcripts with kappa coefficients below .7.

During the second phase, we will assess the potential impact of the system by contrasting the beliefs of the participants from the two arms, across the constructs in the conceptual framework and the emerging categories identified during the first phase. This comparison will allow us to propose hypotheses about the underlying mechanisms that operate over the implementation process, as well as the effects of using each tool. Specifically, those barriers and facilitators that are not present in one scenario or to which the subjects refer to with contrasting emphasis will form the basis of hypotheses about the impact of the DSS in comparison with the printed materials. Barriers and facilitators that seem to have the same emphasis in both scenarios will form the basis of hypotheses concerning determinants not related to the implementation tool.

Additionally, the analysis in both phases will consider the type of center the subject belongs to (ie, urban or rural), the number of years the subject has used smartphones, and whether the subject was provided with a smartphone to participate in the study.

### Protection of Human Subjects

We do not expect that participating in the study will put the subjects at a greater risk of harm or discomfort than what they encounter in their daily working life. Therefore, under the Federal Policy for the Protection of Human Subjects [33] and the Colombian regulation [34], it could be considered that this research does not involve more than minimal risk.

The process of informed consent to participate will start before collecting any data and will continue through the entire study. Before being interviewed, the participants will have time to read the consent document and ask questions about the study goals, the procedures in which they will be involved, and the measures undertaken to protect their rights as human subjects. At any time during the study, the participants will be free to withdraw their consent to participate and leave the study, stop the recording, order the destruction of any audio records they have participated in, and terminate or reschedule an interview.

The interviews will be scheduled at the participants’ convenience, with special attention given to not disrupting clinical practice. Audio records and transcripts will be securely stored and will not be shared with persons other than the 2 researchers involved in the analysis. Additionally, no publication will mention the participants’ names or the names of the study sites. Although we will offer an economic incentive for participation, its value is small in comparison to the participants’ monthly income. Therefore, we do not expect the compensation to compel subjects to participate. Finally, this research protocol has been approved by the institutional review boards of the Pontificia Universidad Javeriana in Bogotá, Colombia and the University of Pittsburgh in Pittsburgh, PA, USA.

## Results

### Intervention Design

We designed and implemented the mobile DSS, following the process presented in Figure 2. During the first stage, we had four meetings, with the participation of 1 engineer (with graduate-level training in biomedical informatics) and 2 pulmonologists who participated in the guideline development. These sessions resulted in a complete algorithm, including the formal definition of risk factors, signs, and symptoms. Some of these definitions were tacit in the text (eg, the number of years spent as a passive smoker that constitutes a risk factor). The algorithm also included previously undefined end points (eg, how patients with a risk factor but no symptoms should be managed).

**Figure 3:**
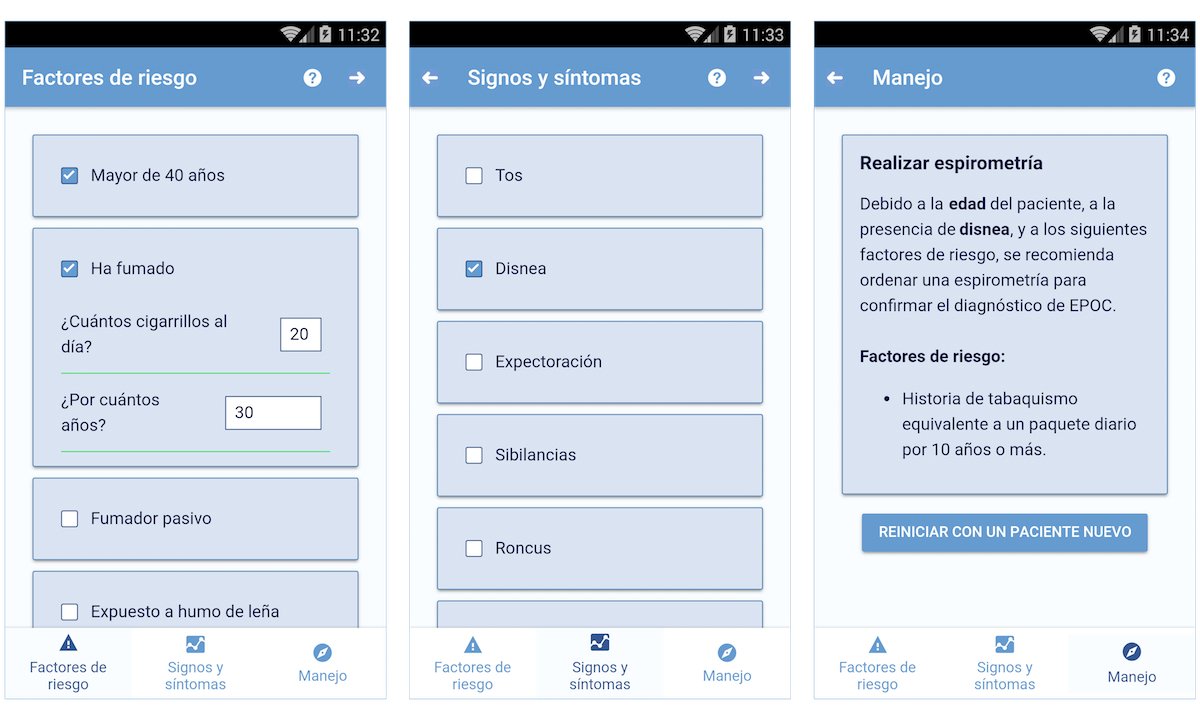
Screenshots of the app.

During stage two, we implemented the algorithm in the form of a mobile app. To fulfill the requirement of being cross-platform (ie, being able to run on iOS, Android, and Windows Mobile), while reducing the development effort, we programmed the app using the IONIC 2 platform [35].

As presented in Figure 3, the app consists of three sections. In the example, the user is stating that the patient is older than 40 years (in Spanish: “Mayor de 40 años”), and has smoked approximately 20 cigarettes per day for 30 years (in Spanish: “¿Cuantos cigarrillos al día? 20” and “¿Por cuántos años? 30”).

The first two sections contain checklists. The first captures the information related to risk factors, while the second concerns signs and symptoms. Some items include second level questions. For instance, when the user indicates that the patient has smoked (in Spanish: “Ha fumado”), the app asks how many cigarettes and for how many years. Finally, the third section presents the algorithm end points depending on the information entered. In the example, due to patient’s age, his smoking history and the presence of dyspnea, the app recommends the user to order a spirometry test.

### Next Steps

We are in the process of harmonizing the concepts from the contributing conceptual frameworks [27-30]. Data collection is expected to occur during 2018, with the first phase of analysis running in parallel. The second phase is scheduled to conclude in July 2019.

## Discussion

Dissemination and implementation of CPGs is recognized as a challenging problem [1], and sometimes, a moving target [2]. Many governments and organizations are turning their attention to mobile DSSs hoping to find an effective, affordable, and scalable solution [9,14,36]. However, there is little evidence to support that expectation [16,17].

Our study will provide qualitative evidence about the potential effects of mobile DSSs in the context of a middle-income country. Furthermore, since the study design considers environments with constraints in informatics resources, our results may be used to inform decisions in low-income countries.

Finally, several authors have highlighted the need for theory-based research about the determinants of the effectiveness and adoption of these interventions [18-20]. Being based on a comprehensive conceptual framework, which considers determinants of behavioral change and technology acceptance, our study will provide actionable evidence that may be translated into concrete programs to promote practice change.

## Abbreviations

CPG: clinical practice guideline
D&I: dissemination and implementation
DSS: decision support system
mHealth: mobile health
TDF: Theoretical Domains Framework
UTAUT: Unified Theory of Acceptance and Use of Technology
